# A Narrative Review on the Human Health Effects of Ambient Air Pollution in Sub-Saharan Africa: An Urgent Need for Health Effects Studies

**DOI:** 10.3390/ijerph15030427

**Published:** 2018-03-01

**Authors:** Eric Coker, Samuel Kizito

**Affiliations:** 1 School of Public Health, University of California-Berkeley, Berkeley, CA 94704, USA; 2 College of Health Sciences, Makerere University, Kampala, Uganda; somekizito@yahoo.com

**Keywords:** ambient air pollution, epidemiology, narrative review, sub-Saharan Africa

## Abstract

An important aspect of the new sustainable development goals (SDGs) is a greater emphasis on reducing the health impacts from ambient air pollution in developing countries. Meanwhile, the burden of human disease attributable to ambient air pollution in sub-Saharan Africa is growing, yet estimates of its impact on the region are possibly underestimated due to a lack of air quality monitoring, a paucity of air pollution epidemiological studies, and important population vulnerabilities in the region. The lack of ambient air pollution epidemiologic data in sub-Saharan Africa is also an important global health disparity. Thousands of air pollution health effects studies have been conducted in Europe and North America, rather than in urban areas that have some of the highest measured air pollution levels in world, including urban areas in sub-Saharan Africa. In this paper, we provide a systematic and narrative review of the literature on ambient air pollution epidemiological studies that have been conducted in the region to date. Our review of the literature focuses on epidemiologic studies that measure air pollutants and relate air pollution measurements with various health outcomes. We highlight the gaps in ambient air pollution epidemiological studies conducted in different sub-regions of sub-Saharan Africa and provide methodological recommendations for future environmental epidemiology studies addressing ambient air pollution in the region.

## 1. Introduction

Recent global burden of disease modeling suggests that low-income countries of sub-Saharan Africa (SSA) suffer the highest burden of disease and premature death attributable to environmental pollution [1]. According to the World Health Organization (WHO), ambient air pollution (AAP) levels exceed recommended limits for as much as 92% of the world’s population [2], and, compared to all other forms of environmental pollution (e.g., water, soil, and occupational), air pollution causes the largest number of environmental pollution-related deaths [1]. As a result, the new sustainable development goals places high priority on reducing the impacts of ambient air pollution on non-communicable diseases [3].

In SSA, there is a paucity of AAP epidemiological studies. In addition, the vast majority of air pollution epidemiological studies in the region are on indoor air pollution (IAP) from household use of cooking fuels. Consequently, the literature regarding the health effects of AAP comes from studies conducted mostly in North America and Western Europe. For instance, the most recent report on the global burden of disease attributable to air pollution derived their AAP burden estimates using integrated exposure–response functions based on data entirely from North American (US and Canada) and European epidemiologic studies [4]. Several recent systematic reviews and meta-analyses on the health effects of AAP for heart failure [5,6], hypertension [7], pneumonia [8], and asthma [9], included studies from every other continent except SSA. Importantly, there is very good reason to suspect that North American- and European-based exposure–response functions from air pollutants, such as PM_2.5_, may not be readily generalized to such low-income settings in much of SSA, suggesting the possibility that the health burden attributable to AAP may be underestimated in the region.

There are important population-level vulnerabilities and individual-level susceptibilities in SSA which may influence exposure-response relationships of air pollution. Vulnerabilities include multiple overlapping socioeconomic risk factors, access to quality health care, and co-prevalence of chronic and infectious diseases (e.g., tuberculosis (TB) and HIV). Secondly, from the sparse air quality monitoring data that are available for the region, average mean levels of PM_2.5_ in some urban areas have been measured at an order of magnitude higher than levels measured in North American and European urban AAP epidemiology studies. Hence, there is greater uncertainty in the magnitude of health effects at high end air pollution exposures since so few studies have been conducted in the most polluted regions [4]. Thirdly, since air pollution is a complex mixture of gases and particulates that is largely determined by the sources of air pollution, there are likely to be substantive differences in air pollution sources and thus important differences in air pollution components between higher income countries and the lower income countries of SSA. Therefore, the toxicity of air pollutants such as PM_2.5_ may be inherently different in ways that place even greater uncertainty in understanding the population health impact of air pollution in the region. It is therefore important to understand the current state of the literature regarding air pollution epidemiologic studies conducted in SSA to date, and to further identify critical gaps in the literature that should help guide future AAP epidemiological research in SSA; a region that lacks adequate air quality monitoring networks and the political will to address air pollution and its impacts [3], and that also has important population-wide vulnerabilities.

In this review article, we summarize the state of the literature on AAP epidemiologic studies throughout the SSA region. To highlight important gaps in the existing literature, and thus a path forward for future studies in the region, we emphasize the following themes in the review: (1) the regional distribution of AAP epidemiology studies; (2) the types of ambient air pollutants measured; (3) the health outcomes examined; and (4) the observed direction of acute and chronic health effects. This review synthesizes knowledge about a major region of the global population that is severely under-studied in the AAP epidemiology literature and thus addresses an important environmental justice issue in global health [1].

## 2. Methods

### 2.1. Systematic Review

Our systematic review of the published literature focused on original research articles in English language peer-reviewed scientific journals. Search terms were used in a manner that coupled “air pollution” and <country> together or, alternatively, “air quality” and <country> together, with the second search term a designated country (*N* = 48) within SSA. In other words, we searched for all studies within SSA with “air pollution” or “air quality” in the text of the article and each search was performed separately for each individual country. We used identical search terms in PubMed, EMBASE, and Google Scholar databases. There was no restriction based on online data base searches in terms of time period of the study or the publication date. From the resultant searches, we reviewed all relevant abstracts and selected out all of those studies that indicated air pollutants were measured as a basis for exposure assessment to air pollution in the study population and where statistical analyses were undertaken to test associations between ambient air pollution measurements and any health outcome of interest and where exposure–response relationships for specific pollutants are reported. We did not restrict the analysis based on any sub-groups (e.g., age, sex, or urban or rural) or by study design. Since IAP and its health effects have been reviewed extensively already, we excluded studies that investigated IAP as the only exposure of interest instead of AAP. In our review of the literature, we decided to only report on those studies with actual air pollutant measurements to highlight the importance of more studies that actually measure air pollutants to derive exposure–response relationships. In addition, since we are strictly interested in epidemiological studies and where pollutant measurements are used to derive an exposure–response relationship, studies were not included in this review if they only related AAP measurements with biomarkers of exposure (e.g., exhaled or urinary markers) nor were they included if factors such as geographic location or occupation were used as air pollution exposure proxies.

**Figure 1:**
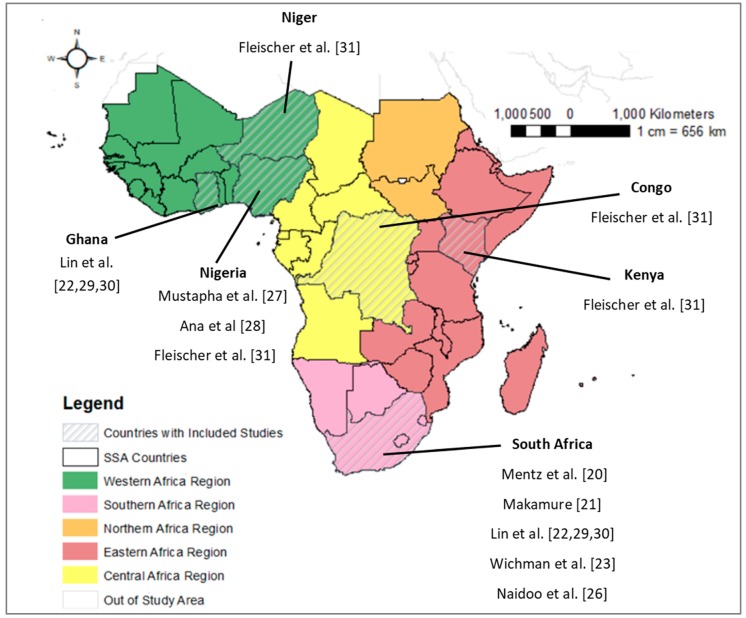
Map of study area and locations of individual-level AAP epidemiology studies. Countries with individual-level AAP epidemiology studies are indicated with diagonal grey lines and the respective study author and citation number provided for each studied country.

In our review, we defined SSA countries as those classified as such by The World Bank [10]. Hence, in our review, we searched for the SSA countries mapped in Figure 1.

### 2.2. Descriptive and Narrative Analysis

Our analysis of the reviewed studies is mostly qualitative because we only provide descriptive summaries across all studies and stratified by key aspects of the different studies. Hence, we provide tabular and graphical summaries to help guide the narrative of this review in a focused and consistent manner. We summarized the studies by the following key themes: (1) the regional distribution of AAP epidemiology studies; (2) the types of ambient air pollutants measured; (3) the health outcomes examined; and (4) the observed direction of acute and chronic health effects. We also provide summaries of other subtle, yet important, aspects of the included studies, such as time trends, the study designs, and sub-populations studied. We also review health risk modeling considerations, such as statistical methods used to test exposure–response associations, whether IAP was controlled for in the studies, or whether effect modifiers such as social class, education-level or smoking were considered, or whether a multi-pollutant modeling framework was applied in the analyses.

We did not perform a meta-analysis as part of our systematic review because we felt such an analysis was not warranted given our motivations of the review, the fairly small number of studies, and given that our review is not focused on any single disease or outcome [4]. While our review was performed systematically but with a narrative and qualitative analysis, we do not argue that the currently available literature precludes other researchers from conducting a meta-analysis, but rather this work represents a jumping-off-point for such future efforts.

## 3. Results

**Figure 2:**
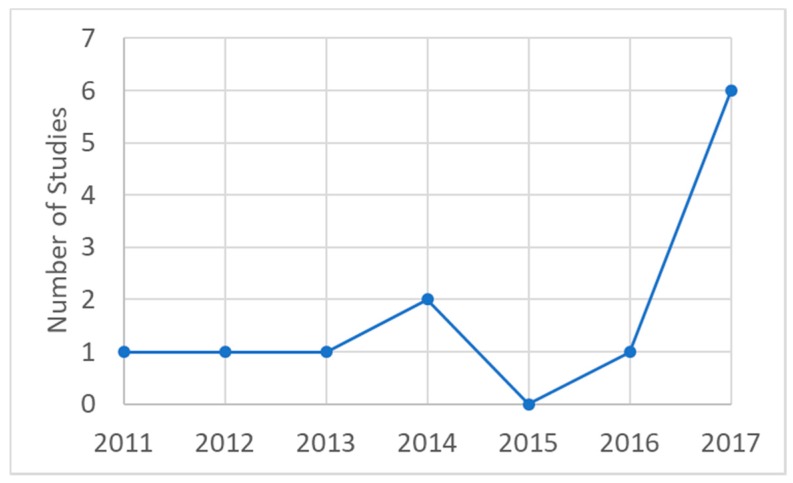
Total number of published ambient air pollution (AAP) epidemiology studies included in the review by year (x-axis).

A total of twelve studies were identified that satisfied our inclusion criteria of studies that derived exposure-response relationships (for any health outcome) using AAP measurements. A detailed summary of the included studies is presented in the Supplementary Materials (Table S1). Remarkably, all of the included studies have been published just within the past seven years (Figure 2) and more than half published in the last two years. In the course of our review, we identified a number of other AAP epidemiological studies from SSA [11,12,13,14,15,16,17,18,19] that did not fit our strict inclusion criteria but are worth noting. While these other studies did not assign air pollution exposure from measurements to derive exposure-response relationships, but instead assigned AAP exposures using proxies such as occupation (e.g., a variety of transportation workers or street vendors), proximity to air pollution sources or intensity of sources, biomarkers (e.g., exhaled CO), perceived air pollutant levels, or comparisons of an air pollution “exposed” region versus a relatively “unexposed” region, each of these AAP exposure-proxy studies show strong and consistent positive associations for health risks such as chronic respiratory, cardiovascular [11,12,13,14,15,16,17,19] or adverse birth outcomes [18]. Since these other exposure-proxy studies are important and may be of interest to readers, a brief summary of these other studies is presented in Table S2 in the Supplementary Materials.

## 4. Regional Patterns of Studies

We observed a substantial disparity in terms of the regional patterns of where air pollution epidemiological studies have been carried out in the region. For instance, of the twelve air pollution epidemiology studies included in the review, 10 tested individual-level AAP exposure-response relationships and these were all carried out in just six countries total (Democratic Republic of Congo (DRC), Ghana, Kenya, Niger, Nigeria, and South Africa). Moreover, of the twelve included studies, three-quarters were carried out in South Africa (*N* = 9, five of which are multi-country studies), with the city of Durban, South Africa comprising 25% of the twelve studies. Of the two studies that included all of the other SSA countries, each one is a multi-country study with an ecologic country-level or region-level design. All of the study populations in the country-specific studies focused on urban populations.

## 5. Summary of Study Outcomes

**Figure 3:**
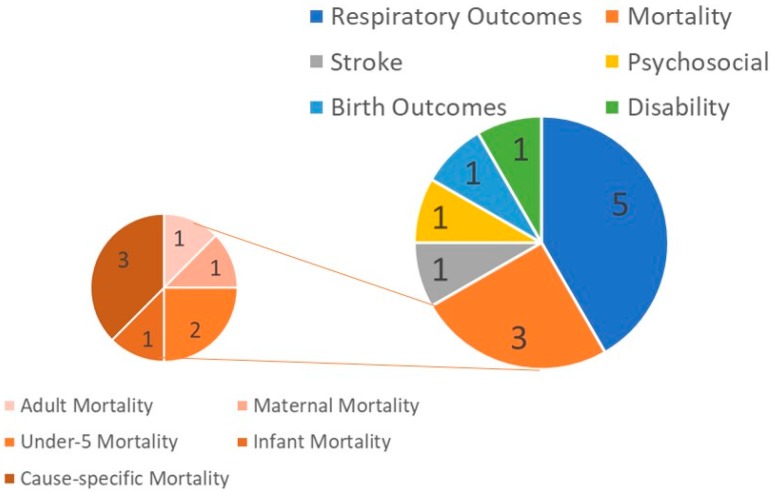
Distribution of types of study by health outcome categories. The smaller pie chart represents the number of mortality outcomes examined within the three different mortality studies identified in our review of the literature.

**Figure 4:**
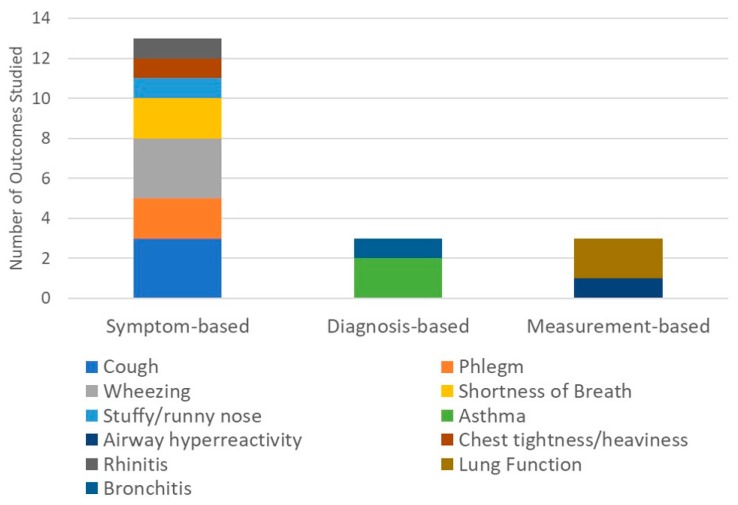
Distribution of types of respiratory outcomes examined for those classified as respiratory studies.

A graphical summary of the study outcomes assessed in the included studies is presented in Figure 3. More than half of the twelve reviewed studies focus on respiratory outcomes or mortality (*N* = 5 and 3, respectively), with the balance focused on stroke, psychosocial (depression), adverse birth outcomes (low birth weight and preterm), and disability. Of the three mortality studies, one is focused on cause-specific mortality (respiratory, cardiovascular, and cerebrovascular) and the other two are focused on population sub-groups (infant, under-five, adult, and maternal mortality). Figure 4 further summarizes the respiratory effects studies by highlighting the types of respiratory outcomes assessed. As indicated in Figure 4, symptom-based respiratory outcomes predominate, followed by diagnosis- and measurement-based respiratory outcomes. Of the symptom-based outcomes, the most common outcomes studied are cough and wheeze (likely because several studies used the International Study of Asthma and Allergies in Childhood questionnaire). Asthma and bronchitis have been studied only twice and once, respectively. Of the biologic measurement-based outcomes, two studies assessed lung function and one evaluated airway hyperreactivity.

## 6. Acute Health Effects Studies

We identified a total of six AAP epidemiologic studies that considered acute health effects. Two respiratory health effect studies considered a range of upper and lower respiratory outcomes (all in children), one study considered stroke, and three other studies considered mortality outcomes. However, we note that only half (*N* = 3) of these studies assigned short-term (acute) exposure from AAP measurements to derive exposure–response relationships, while the other three studies assigned exposure using long-term concentrations (e.g., annualized or three-year averages). In addition, two of the three studies that used long-term concentration exposure data may further be classified as “ecological” designs since health outcomes and pollutant exposures were aggregated to large spatial units (e.g., country-level or region-level) rather than studying exposure–response relationships at the individual-level. Two of these studies were prospective designs and both were conducted in Durban, South Africa. Each of these six AAP acute health effects studies are summarized in turn.

### 6.1. Prospective Studies

A panel study from South Africa recruited school-aged children (*N* = 423) from Durban schools [20]. This study measured PM_10_, SO_2_ and CO at the participant’s school and utilized NO_2_ and NO ambient air measurements collected from eight government air monitoring sites as well as O_3_ ambient air measurements collected at two other government air monitoring sites. Both single-pollutant and bi-pollutant associations were investigated for several acute lower respiratory outcomes. Self-report logs of acute respiratory symptoms were recorded multiple times during the day. Acute symptoms included cough, wheezing, shortness of breath (SOB), chest tightness or heaviness. Significant associations were observed for each air pollutant, with all but one resulting in adverse effects. Cough, SOB and chest symptoms were all higher with increasing PM_10_, SO_2_, NO_2_, and NO concentrations. Wheeze was also higher with increased NO_2_ but lower with higher NO. The strength of these associations for each individual pollutant depended on the number of day lags, with cough most consistently stronger for same day or five-day lags and chest tightness consistently strongest on same day exposure. There was no clear pattern for the other outcomes with respect to lag-days. This study did not, apparently, collect important information related to biomass fuel use at participants’ homes.

A prospective cohort study [21], also carried out in Durban, South Africa, tested the interaction between daily pollutant levels (SO_2_, PM_10_, NO_2_, and NO) and CD14 cell genetic polymorphisms on within-day changes in lung function. The study participants were school-aged children (7–9 years of age) (*N* = 71). Forced expiratory volume in 1 s (FEV1) was measured repeatedly every two-hours for three-week periods during different seasons of the year to determine within-day changes in lung function. Continuous air pollutant measurements occurred at the schools, and in some cases, off of school grounds, with exposure assignment based on daily average concentrations from each school or nearest monitor to the school. Generalized estimating equations (GEEs) related exposure–genetic polymorphism interaction terms with daily changes in lung function. Although none of the pollutants were significantly associated with lung function alone, when analyses were stratified by CD14 polymorphism status, only the CD14 CT/TT polymorphism was associated with daily lung function decrements with increasing NO_2_ and NO daily exposures. This significant interaction was consistent across one-, two-, and five-day lag exposures for both pollutants. The most important contributions from this study is its prospective design and the consideration of individual-level susceptibility using a biologically plausible polymorphism in a gene related to cellular immunity and asthmatic symptoms.

### 6.2. Cross-Sectional Studies

A cross–sectional study [22] utilized household survey data collected between the years 2007 and 2010 in six low- and middle-income countries, which included two SSA countries (South Africa and Ghana). This study tested the association between long–term PM_2.5_ and self-report of stroke in the past 12 months in adults (*N* = 45,625). PM_2.5_ exposure assessment was estimated by deriving three–year average from satellite aerosol optical depth (AOD) measurements (without ground-level measurements). A logistic regression multilevel model resulted in significant positive associations for PM_2.5_ and stroke. Statistically significant effect modification was observed for smoking status, physical activity, and fruit and vegetable intake. These interactions were observed to be in the hypothesized direction in some cases (e.g., fruit and vegetable intake mitigated PM_2.5_ stroke effects and effects were strongest among those with high physical activity), but interactions were in opposite directions than expected for others (e.g., PM_2.5_ effects were strongest in never smokers—possibly due to uncontrolled confounding from environmental tobacco smoke). This study offered several important contributions compared to other studies reviewed; in particular, the authors accounted for IAP sources in the home (such as polluting cooking fuels) and evaluated PM_2.5_ effect estimates for effect modification by multiple individual-level biologically plausible risk factors including sex, age, smoking, physical activity, and nutrition (fruit and vegetable intake). Another important contribution of this study, compared to other reviewed studies that used nearest monitor exposure metrics, is the use of satellite derived AOD measures of PM_2.5_ exposure. Satellite derived measures provides spatially resolved estimates that may reduce exposure misclassification in epidemiological studies. An important limitation in this study is a reliance on long-term concentrations for acute stroke events; although air pollution and stroke likely have chronic exposure etiology.

The three remaining acute health effects studies are all mortality studies, two of which are ecological designs and one a case-crossover design. The case-crossover study (*N* = 149,667), from Cape Town, South Africa, tested the year–round and seasonal associations between daily NO_2_, SO_2_, and PM_10_ with respiratory disease (RD) mortality, cardiovascular disease (CVD) mortality, and cerebrovascular disease (CBD) mortality between 2001 and 2006 [23]. Pollutants were measured hourly at three government air monitoring sites and daily exposures were assigned to the population of Cape Town by averaging across the three monitoring sites to derive daily average values. Daily mortality data, with their corresponding causes of death codes, came from the City of Cape Town mortality records. For NO_2_, statistically significant positive year-round associations were observed for CVD and CBD mortality. PM_10_ and SO_2_ showed a significant positive year-round association for CBD and CVD mortality, respectively. RD mortality resulted in significant positive associations with NO_2_ and PM_10_ exposures during warmer times of the year only. No positive associations were seen between study pollutants and any cause-specific mortality outcomes during the colder time of the year [23]. There are several notable strengths in this Cape Town study. The authors tested for effect modification by sex, age, and distance from monitors (distance from monitor showed significant interaction effects). In addition, this is the only study to have applied a case-crossover design—where individuals serve as their own controls—which is a particularly compelling approach for examining acute health effects from AAP. This was also the only study to explore the potential seasonal trends in pollutant associations.

The two ecological mortality studies are quite different than the Cape Town study primarily because they use either country-level or sub-region-level annual PM estimates and annual mortality estimates. This type of study design has notable weaknesses, such as being susceptible to ecological fallacy and applying long-term exposure estimates to what is essentially an acute health outcome (mortality). Despite these glaring limitations, each of these ecological studies makes unique contributions to the literature on AAP health effects in the region. The study by Aliyu and Ismail [24] used data from 35 African countries between the years 1995–2001 and explored sex-specific relationships between country-level long-term PM_10_ and CO_2_ emissions data and rates of adult mortality, infant mortality, and under-five mortality. The authors analyzed the data using an econometric generalized method of moments model (using autoregressive terms on the outcome and exposure) and found that PM_10_ and CO_2_ emissions were associated with increased male and female adult mortality (though marginally higher in females) and increased under-five and infant mortality. The analysis evaluated effect modification by country-level governmental effectiveness and found that better government effectiveness was associated with lower AAP-mortality risk, suggesting that good governance can play a role in mitigating the health impacts of AAP. The other ecological study, by Owili et al. [25], took an especially novel PM_2.5_ source apportionment approach by using spectral aerosol optical depth satellite data to discriminate PM_2.5_ source-specific concentrations as well as source-specific exposure–response relationships with under-five mortality and maternal mortality. The authors used a parametric generalized linear and additive mixed-effect model with natural cubic splines (GLMM + NS) and a Poisson link function as the main analysis for inference (they also supplemented with an alternate non-parametric regression analysis). From the main GLMM + NS analysis, pollutant concentrations for both biomass and anthropogenic PM_2.5_ sources were significantly associated with higher under-five and maternal mortality (results were mostly consistent with the non-parametric analysis) (between the years 2000–2015). Conversely, dust PM_2.5_ and PM_2.5_ from a mixture of sources were both associated with lower under-five and maternal mortality, although there was some conflicting evidence from non-parametric analytic results. While the exposure and outcome data were aggregated at the sub-region level for Africa (Central Africa, Eastern Africa, Northern Africa, Southern Africa, and Western Africa), which is a severe limitation, this study is nonetheless important. The apparent ability to separate out PM_2.5_ effects from different sources, by discriminating sources using the spectral nature of light particle scattering, is the most noteworthy contribution of this study [25]. There are clear regional variations in PM_2.5_ sources which may impart spatial variability in the toxicity of airborne PM_2.5_. This appears to be expressed by these findings; with biomass and anthropogenic PM_2.5_ sources indicative of higher under-five and maternal mortality risk compared against dust or mixed sources of PM_2.5_ with lower risk.

## 7. Chronic Health Effects Studies

A cross-sectional South Africa study recruited school-aged children (*N* = 341) from Durban schools [26]. This study measured PM_10_ and SO_2_ at the participant’s school and utilized NO_2_ and NO air monitoring data collected from 8 government monitoring sites. Single-pollutant associations were investigated for several chronic lower and upper respiratory outcomes; these included care-giver report on cough, phlegm, bronchitis, wheezing, wheezing with shortness of breath, runny nose, itchy and watery eyes, and asthma. Biologic measurement outcomes included airway hyperreactivity (methacholine challenge tests), pulmonary function measurements, and skin allergy testing. There were no observed associations between air pollutants and study outcomes except only that SO_2_ was significantly associated with increased odds of airway hyperreactivity. This was the only reviewed study that examined biologic measures of airway hyperreactivity. While this study did collect information on biomass fuel use (an important risk factor for chronic respiratory illness), this exposure was curiously omitted from the multivariate regression model results, which may partially explain the consistent null findings for most exposure–response relationships.

Another cross-sectional study [27], conducted in 2004 in the Warri region of Nigeria, recruited schoolchildren (ages 7–14 years; *N* = 1397)) to examine associations between measurements of traffic-related air pollution and several chronic respiratory symptoms and asthma. Survey questions from the International Study of Asthma and Allergies in Childhood questionnaire were used to determine lower respiratory symptom prevalence and asthma prevalence in the participants. Despite a lack of routine air monitoring in the study area, researchers conducted brief air sampling at the study schools, sampling for carbon monoxide (CO) and PM of varying fraction sizes. Measured CO and PM air concentrations were then combined with indicators of nearby traffic activity in a principal component analysis. The top three principal components were used to assign exposures to participants from study schools which were in turn used as inputs in the logistic regression analyses. Principal component 1 (comprised of traffic pollution) was associated with higher phlegm while principal component 3 (comprised of fine particulates related to truck traffic) was also associated with higher phlegm. No other outcomes showed significant associations. Importantly, this study did control for household IAP sources as determined from study questionnaires. The results from this analysis may be considered a multi-pollutant analysis because it combined concentration data on more than two pollutants, including CO and various PM fraction sizes. Therefore, this study represents the only one in the region to have presented results from a multipollutant analysis, which makes it a particularly important contribution to the literature. By combining information on multiple air pollutants with indicators of traffic-related pollution, the authors were able to demonstrate the importance of traffic-related air pollution on children’s lower respiratory health.

Another cross-sectional study from Nigeria [28], conducted in 2008 in the city of Ibadan, investigated the relationship between PM_10_ concentrations and measures of lung function among selected study participants (*N* = 140, ages 15–65). Participants were non-smokers, between the ages of 15–65 years, had no family history of respiratory disease and lived at least for three years in the study area. Air sampling for PM_10_ took place over the course of three months in 2008 at time intervals intended to represent air quality during the morning and late afternoon times. Spirometry was performed on study participants to record observed forced expiratory volume in one second (FEV1) and participant body mass index was measured to derive a predicted FEV1 and to calculate percentage predicted FEV1 (% FEV1). Spearman rank test correlation was performed to observe associations between PM_10_ concentration and lung function measures. The analysis revealed that only the observed FEV1 measure was significantly associated with lower lung function [28]. A major limitation of this study was a lack of multivariate analysis leaving the results prone to bias from confounding. Other limitations include a reliance on sampling during certain time-intervals each day, which may not be entirely representative of chronic daily air pollution exposures thus increasing the likelihood of exposure misclassification that would bias findings towards the null. However, this represents the only study to have related AAP measurements with lung function measurements, making it a unique contribution to the literature on AAP health effects studies in SSA.

Two additional cross-sectional studies by Lin et al. [29,30] used household survey of six low and middle income countries, which included data from South Africa and Ghana as well as countries from Asia and Latin America between the years 2007 and 2010, carried out a multilevel regression analysis to determine the association between PM_2.5_ and depressive symptoms (*N* = 41,785) [29] and disability score (*N* = 45,625) [30] as chronic outcomes. PM_2.5_ exposure assessment was estimated by deriving three-year average from satellite AOD measurements (without ground-level measurements). Estimates of PM_2.5_ concentration was positively associated with depressive symptoms [29] and disability score [30], controlling for household IAP indicators. Interestingly, smoking was shown to be an effect modifier for depression, with an observed additive interaction between PM_2.5_ and smoking on depression. In our review of the current literature, these are the only studies that examined a psychosocial or disability outcome.

The only study to research air pollution associations with adverse birth outcomes in SSA is also a multi-country study, using data on births collected in 2004 and 2005 from study clinics around the world [31]. Among other countries in Asia and Latin America, several clinic field sites in SSA were included in the study; these were Democratic Republic of Congo (DRC) (*N* = 7067), Kenya (*N* = 16,694), Niger (*N* = 4826) and Nigeria (*N* = 6538). This study tested associations between PM_2.5_ exposure estimates during pregnancy and preterm birth and term low birth weight. Length of gestation and birth weight were recorded at birth and collected from each study clinic. PM_2.5_ exposure during pregnancy was determined with AOD satellite-derived data for 2001–2006 and were combined with vertical column aerosol values from a chemical transport model (these are essentially the same estimates as in Lin et al. [22,29]). Maternal exposure was estimated within a 50-km buffer surrounding each mother’s respective delivery clinic and seasonal adjustments were applied to the long-term pollutant estimates to estimate exposure during the last month of each pregnancy at each mother’s delivery clinic 50-km buffer. Generalized estimating equations (GEE) were implemented in the study to account for clustering of birth outcomes within clinic, with confounder adjustment for maternal and infant factors in one model and country-level factors added in a second model. Only term low birth weight was significantly associated with PM_2.5_ exposure levels during pregnancy, at the two highest exposure-level quartiles, although the association was slightly attenuated after controlling for country-level confounders (albeit still statistically significant). Country-level estimates of associations could only be estimated for India and China due to limitations of sample sizes from the other countries, hence no country-level estimates were determined for SSA countries. The primary strength of this multi-country study is the uniform collection of birth and covariate data as well as the exposure estimation procedure across the study sites. However, this study relied heavily on spatial variation in exposure-levels while temporal variability in exposure-levels were extremely limited by way of simple seasonal adjustments. In addition, exposure assignment assumed mothers resided within a 50-km buffer of the health facility, further increasing the risk of exposure misclassification.

## 8. Discussion

Our review of the AAP epidemiology literature from SSA revealed a very few number of studies relating air pollution measurements with health outcomes as well as extreme geographic disparities in terms of where studies have been conducted. For instance, most of the AAP epidemiology studies were concentrated in a single country (South Africa). While only twelve studies were identified that fit our inclusion criteria, most studies varied in terms of design and the nature of the outcome. On the other hand, each of the studies made a unique and potentially valuable contribution towards not only understanding the relationship between air pollution and health in the region, but also towards guiding future directions for AAP epidemiological studies in the region. From our review of the literature in regards to air monitoring for epidemiology studies in SSA, we identified several important gaps.

### 8.1. Gaps in Ambient Air Pollution Monitoring and Exposure Estimation

First and foremost, the only region with an adequate air monitoring network that may be leveraged for health effects studies appears to be in South Arica [3]. This likely played a key role in our determination that South Africa tended to have the highest quality studies, whether it be for testing acute mortality or respiratory outcomes or chronic health outcomes in relation to AAP exposure. Studies from South Africa were able to test associations for several different particulate and gaseous pollutants, whereas studies from Nigeria and Ghana were typically relegated to either a single pollutant or a composite of convenience air pollutant measurements and traffic indicators. Hence, exposure characterization was the most consistent between the South Africa studies and produced the most time-resolved data for acute health effects studies and prospective designs, whereas exposure characterization in the Nigeria studies varied considerably and were not readily comparable.

While air monitoring networks may be emerging in other SSA countries, it is clear that effort should be put towards developing air monitoring networks that can facilitate epidemiology studies throughout the region. We note that low-cost air monitoring instruments have recently been validated and shown to be effective compared against established government air monitoring instruments in the US [32]. In addition, low-cost passive air samplers for NO_2_ have been validated in Durban, South Africa [33]. Low-cost air samplers may offer a viable alternative compared with costly and complex air monitoring instruments used in higher income countries.

Another glaring gap in the region’s AAP epidemiology literature is a lack of what is now-conventional land use regression (LUR) modeling. Although satellite derived methods have been used in at least four of the reviewed studies, there is still a limited range of pollutants that can be estimated with this method and such exposure metrics are most suitable for chronic exposure and chronic outcome studies. LUR models could be most helpful for exposure assessment in smaller-scale sub-regional AAP epidemiology studies that require spatially- and time-resolved estimates of exposure, especially for acute health effects studies. We identified at least one study on the horizon, in Western Cape, South Africa, that will implement state-of-the-art LUR modelling to derive exposure estimates in a respiratory health effects study [34].

In the near-term, given the lack of air quality monitoring networks in the region and the high cost of state-of-the-art sampling instrumentation, we recommend the deployment of low-cost and validated air samplers combined with GIS data collection. Such an approach may be leveraged for LUR exposure estimation for chronic or acute health effects studies. Satellite-derived estimates of exposure should also be leveraged in studies where ground-level measurements are not feasible. However, we note there is a general need for ground-level monitoring to validate satellite-derived concentration estimates in the region.

### 8.2. Health Outcome Gaps

Another important gap that emerged from our review of the literature is a lack of studies that evaluated health care records for cause-specific outcomes (e.g., hospitalizations or hospital visits) or mortality data with the appropriate spatial resolution. Only one of the reviewed studies evaluated cause-specific mortality and none of the studies examined cause-specific hospitalizations or hospital visits. Of the respiratory health studies reviewed, only two studies examined a specific diagnosis (asthma and bronchitis) and these were both self-reported diagnosis instead of documented cases. Moreover, most of the mortality studies we reviewed applied an ecological design with country-level mortality data. Without spatially resolved AAP measurements spatially linked with individual outcomes, findings will continue to be prone to ecological fallacy. Where available, effort should be made to conduct studies that collect hospitalization and mortality data coupled with spatial information that enable data linkage studies for within and between region studies. More studies on cause-specific mortality, hospitalizations, and hospital visits are critically needed to provide insights into the population health impact of AAP in the region. Notably, children comprised the study populations for all of the non-mortality-based and country-specific studies; suggesting a need for more health outcomes studies that include elderly populations in future studies.

In terms of the range of outcomes examined in the included studies, there are some important holes that need to be addressed. Only one study included in our review investigated adverse birth outcomes. Given the epidemiologic evidence for associations between AAP and adverse birth outcomes and the high rates of adverse birth outcomes in SSA, such as low birth weight and preterm birth, there is a need for more such studies. As already mentioned, diagnosis- and measurement-based outcomes are rarely studied in the region. In addition, other respiratory outcomes yet to be explored, in relation to AAP, include pneumonia and TB, which also deserve greater attention given the large burden of these respiratory diseases in the region.

There is also a relatively poor understanding of the AAP sources throughout the region. Like any other part of the world, the sources of AAP in SSA are a mixture of a variety of natural and anthropogenic sources. However, the relative contribution of specific sources of PM pollution is likely to be in stark contrast to the higher income countries of North America and Europe (where most studies have taken place). For instance, of the few source apportionment studies conducted in the region, the predominant sources for PM pollution in Accra, Ghana [35,36] and Kampala, Uganda [37] included biomass burning, heavily-congested traffic on roadways, loose dirt from road surfaces, fish smoking, and trash burning or incineration. Many of these PM sources are not as typical for urban areas of the higher income countries. In addition, vehicle emissions are poorly regulated and the operating conditions of the ubiquitous use of older emitting vehicles is similarly different compared with higher income countries [3]. It is therefore important for future studies to take into account the different and unique sources of urban AAP in the region, particularly the impact of biomass and heavy traffic congestion and roadway dust.

### 8.3. Data Analysis Gaps

Very few studies implemented key analytic elements that should be incorporated into future studies. Only three of the twelve studies controlled for IAP in the home, even though household IAP sources are ubiquitous in the region and the compelling evidence for substantial health effects from IAP in SSA. Since IAP levels are likely to have overlapping health impacts with AAP and likely to be correlated with AAP levels under certain circumstances, there is a clear need to ensure IAP data are collected and incorporated into AAP epidemiologic analyses. On a positive note, at least four of the reviewed studies tested for interaction between AAPs and relevant factors such as smoking, age, sex, season, physical activity, and nutrition. Notably, several of these factors exhibited significant interaction effects, including smoking, warmer seasons, physical activity, and nutrition. These results suggest the need for incorporating effect modification into studies. It is also important to note that none of the reviewed stories explored effect modification by socioeconomic factors like education or poverty, nor was disease co-prevalence (e.g., HIV or TB) explored as an effect modifier. SSA suffers from high poverty and inequality, along with other social stressors and co-prevalence of diseases that can contribute to susceptibility and vulnerability to air pollution impacts on health. There is a critical need to consider these social and comorbidity factors in future AAP epidemiology studies, especially for the rapidly growing urban slum populations of SSA.

For the reviewed studies that measured several pollutants, there was an overall lack of application of a multi-pollutant framework. While this is a well-known and general deficiency in most AAP epidemiology studies, mostly due to the analytic challenges imposed by such a framework, nevertheless future studies should apply a multi-pollutant framework where the data are suited to do so. This may be particularly important for SSA because dust particles and biomass burning comprise a major portion of PM pollution and rapid industrialization and urbanization are co-occurring with these air pollution factors. The study by Owili et al. [25], which showed variation in mortality effects depending on PM source, highlights the importance of applying a multi-pollutant framework. Additionally, in one of the reviewed studies, air pollution associations with RD mortality were shown to be strongest during the warmer part of the year in South Africa [23]. This finding may have important implications with respect to the effects of air pollution in the context of warming trends related to climate change. The role of warmer temperatures to act in synergy with air pollution exposure warrants further research in the region [38].

### 8.4. Geographic Gaps in AAP Epidemiology Studies

The most obvious gap in our review of the AAP epidemiological literature is the extreme disparities in terms of where studies from SSA have occurred. Not a single individual-level AAP epidemiology study was conducted in Northern SSA and just one in Eastern Africa and one in Central Africa. Even within Western and Southern Africa regions, there are extreme disparities, whereby individual-level studies have occurred in a limited range of areas of South Africa and Nigeria. There is a clear need to conduct studies across a more diverse range of urban areas as well as a need for studies in rural areas where biomass burning could be a contributor to poor air quality.

### 8.5. Limitations and Strengths

There are some important limitations and strengths regarding our review. There is a possibility that we missed some published articles not represented in PubMed, EMBASE, or Google Scholar. While we recognize this as an important limitation, we are confident that the themes and gaps we identified in our review of the literature is likely to persist. Another limitation is that we did not quantitatively determine effect sizes across studies using a meta-analysis. Even though our motivation here is to simply provide a narrative review to highlight the current state of the literature, there is still a need for a meta-analysis to quantitatively assess effect sizes and compare these with existing literature.

The most important strength of our review is that it synthesizes the range of AAP epidemiology studies carried out in SSA in a consistent manner to highlight important methodological gaps and geographic disparities in the conducting of such studies. We also provide recommendations on future studies in the region to better ascertain the population health impact of AAP.

## 9. Conclusions

There is a paucity of epidemiological studies regarding ambient air pollution in sub-Saharan Africa, which is worsened by the mal-distribution of the few available studies with most of the studies coming from Southern Africa. More studies are critically needed to provide insights into the population health impact of ambient air pollution in the region; for instance, studies evaluating individual-based and population-based outcomes like cause-specific mortality, hospitalizations, and hospital visits. This review highlights a dire need to improve on the air monitoring networks in sub-Saharan Africa to enable high quality epidemiological studies as a first step in addressing the impacts off ambient air pollution in the region. This can be achieved using the recently validated low-cost air monitoring instruments combined with readily accessible GIS information and methods. This should be coupled with the deployment of data robust analysis techniques, to maximally utilize the few available data. Population susceptibility should also be a focus of future studies, especially regarding the high rates of social deprivation and co-morbidities like HIV and TB. Despite the limited number of studies meeting our strict inclusion, we have been able to demonstrate the profound effect of ambient air pollution on both the acute and chronic health outcomes in the population in sub-Saharan Africa, a region that is hit by a dual burden of infectious and non-infectious risk factors for lung disease.

## Supplementary Materials

The following are available online at http://www.mdpi.com/1660-4601/15/3/427/s1, Table S1. Tabular summary of studies that met the inclusion criteria. Table S2. Tabular summary of notable studies that did not meet the inclusion criteria.
