# Supplementary material for: A Narrative Review on the Human Health Effects of Ambient Air Pollution in Sub-Saharan Africa: An Urgent Need for Health Effects Studies
Source: Int J Environ Res Public Health. 2018 Mar 1;15(3):427. doi: 10.3390/ijerph15030427 (PMC5876972; doi:10.3390/ijerph15030427)
Supplement: Supplementary file 1 [file ijerph-15-00427-s001.pdf]

Table S1. Tabular summary of studies that met the inclusion criteria.												
**Title and author [reference#]**	Year	Country	Time-frame	Type	Study Population	Health Outcome/s	Exposure variables	Statistical Analysis	Key findings – adjusted Odds Ratios (95%CI) or β (95%CI) or adjusted hazards ratios (HR)	Assessed for interaction with socioeconomic or other risk factors?	Multi-pollutant models?	Controlled for indoor fuel use?
**Acute respiratory symptoms associated with short term fluctuations in ambient pollutants among schoolchildren in Durban, South Africa; Mentz et al. [20][a]**	2017	Durban, South Africa	Not indicated in publication	Longitudinal panel study of children from different schools followed over time	N = 423 school children, 44% African, 23% mixed race, 28% Indian, 6% white	Acute respiratory – cough, wheeze, shortness of breath, chest tightness/heaviness, any symptoms; outcomes ascertained with daily logs recorded bi-hourly	PM10, SO2, NO2, NO measured at 8 monitoring sites and at each school	GEE; 0 – 5 day lags; single lags and distributed lags	PM10 - ↑ cough, ↑ SOB, ↑ chest; SO2 - ↑ cough, ↑ SOB, ↑ chest; NO2 - ↑ cough, ↑ wheeze, ↑ SOB, ↑ chest; NO - ↑ cough, ↓ wheeze, ↑ SOB, ↑ chest	No	Yes, but bi-pollutant model results not presented due to problems from multi-colinearity	No
**Ambient pollution and respiratory outcomes among schoolchildren in Durban, South Africa; Naidoo et al. [26]**	2013	Durban, South Africa	Not indicated in publication	Cross-sectional study of children from different schools	N = 341 school children, 41% black, 22% coulured, 26% Indian, 11% white	Chronic – cough, phlegm, bronchitis, wheezing, wheezing + SOB, Stuffy/runny nose, watery/itchy eyes, asthma, airway hyperreactivity	PM10, SO2, NO2, NO measured at 8 monitoring sites and at each school	Logistic regression	SO2~ airway hyperreactivity HR=2.14 (0.98, 4.66)	No	No	No
**Ambient PM2.5 and Stroke Effect Modifiers and Population Attributable Risk in Six Low- and Middle-Income Countries; Lin et al. [22]**	2017	**South Africa, Ghana**, China, India, Mexico, Russia	2007-2010	Cross-sectional/household surveys	N = 45,625	Stroke – based on self-report over the past 12-months	PM2.5 – yearly averages derived from satellite-derived aerosol optical depth estimates	Logistic regression – 3-level multilevel model	PM2.5 ~ Stroke: OR=1.13 (1.04-1.22)	Yes – sex, age, smoking*, physical activity*, fruit* and vegetable* consumption	No	Yes
**Exposure to air pollution and tobacco**	2017	**South Africa, Ghana**,	2007-2010	Cross-sectional/household surveys	N = 41,785	Depression	PM2.5 – yearly averages	Logistic regression – 3-level	PM2.5 ~ depression symptoms:	Yes – smoking*	No	Yes

**smoking and their combined effects on depression in six low- and middle-income countries. Lin et al. [29]**		China, India, Mexico, Russia					derived from satellite-derived aerosol optical depth estimates	multilevel model	OR=1.09 (1.01, 1.17)			
**Exposure to ambient PM2.5 associated with overall and domain-specific disability among adults in six low- and middle-income countries; Lin et al. [30]**	2017	**South Africa, Ghana**, China, India, Mexico, Russia	2007-2010	Cross-sectional/household surveys	N = 45,625	Disability Score	PM2.5 – yearly averages derived from satellite-derived aerosol optical depth estimates	Linear regression – 3-level multilevel model	PM2.5 ~ WHO Disability Score: β=0.72 (0.22, 1.22)	Yes – sex and age	No	Yes
**Ambient Air Pollution Exposure and Respiratory, Cardiovascular and Cerebrovascular Mortality in Cape Town, South Africa: 2001–2006; Wichmann & Voyi. [23]**	2012	Cape Town, South Africa	2001-2006	Case-crossover using mortality data from local municipal records	N = 149,667 (respiratory disease = 13,439; CVD = 21,569; CBD = 7,594	Mortality – respiratory disease (RD); cardiovascular disease (CVD); and cerebrovascular disease (CBD)	NO2, SO2, and PM10 measured daily at three government monitoring sites	Logistic regression; tested zero – 5-day lags	NO2~CVD: β=3.4 (0.3, 6.6); SO2~ CVD: β=2.6 (0.1, 5.2); PM10~ CBD: β=4.1 (0.4, 8.1); NO2~ CBD: β=8.0 (2.9, 13.4); PM10~RD (warm temps): β=5.5 (1.4, 9.6); NO2~RD (warm temps): β=6.0 (1.0, 11.2); PM10~ CVD (warm temps): β=4.1 (1.0, 7.2); NO2~CBD (warm temps): β=9.0 (2.4, 16.0)	Yes – seasonal temperatures, sex, age, distance from monitors*	No	No
**The effects of air pollution on human**	2016	35 African countries	1995-2011	Longitudinal, population-based panel study (ecological)	Not given in publication	Mortality rate – stratified by male and female adults,	PM10 and CO2 emissions per capita	Generalized method of	PM10 – 0.017% to 0.028% male and 0.024% to	Yes – sex and government effectiveness	No	No

**mortality: does gender difference matter in African countries?; Aliyu & Ismail. [24]**						infants, and under-5 mortality	(source of these data were not well described)	moment regression	0.031%female adult mortality; 0.33% infant and 1.33% under-5 mortality			
**Traffic Air Pollution and Other Risk Factors for Respiratory Illness in Schoolchildren in the Niger-Delta Region of Nigeria; Mustapha et al. [27]**	2011	Nigeria municipalities: Warri, Ebrumede, Effurun, jeddo, Ubeji, Otor-Udu, Owhrode, Ovu, Udu, and Edu	March – June 2004	Cross-sectional survey study	N = 1,397 schoolchildren (7 – 14 years)	Wheeze (past 12 months); Night cough (past 12 months); Phlegm; Rhinitis; Asthma (ever diagnosed)	PM (of various size fractions) and CO. Note: exposure metric based on a principal component analysis that combined air measurements with traffic indicators	Logistic regression	Traffic pollution component ~ phlegm: OR=1.15 (0.89, 1.49); Fine particulates/trucks component ~ phlegm: OR=1.38 (1.09-1.75)	No	Yes – use of PCA may constitute a multi-pollutant framework	Yes
**Outdoor respirable particulate matter and the lung function status of residents of selected communities in Ibadan, Nigeria; Ana et al. [28]**	2014	Ibadan, Nigeria	January – March 2008	Cross-sectional study	N = 140, ages 15-65 years	FEV1 observed and % predicted FEV1 as determined by observed FEV1/predicted FEV1	PM10	Spearman rank correlation test	PM10 ~ FEV1: Pearson correlation= -0.371 (p < .05)	No	No	No
**The Associations between Types of Ambient PM2.5 and Under-Five and Maternal Mortality in Africa; Owili et al. [25]**	2017	All African countries	2000-2015	Ecological study	Not given in publication	Under-five mortality and Maternal mortality	Annual PM2.5 pollution type (biomass, anthropogenic, dust, mixture) as derived from monthly	Generalized Linear and Additive Mixed-Effect models with Poisson link function	Biomass PM2.5~Under-5 deaths: β=0.99 (0.98, 1.02) and Maternal deaths: β=0.94 (0.88, 0.99); Anthropogenic PM2.5~Under-5 deaths:	No	No	No

							spectral aerosol optical depth satellite data to estimate concentrat ions for African sub-region		β=1.01 (1.01, 1.02) and Maternal deaths: β=1.03 (1.01, 1.05); Dust PM2.5~ Under-5 deaths: β=0.98 (0.97, 0.98) and Maternal deaths: 0.93 (0.91, 0.95); Mixture PM2.5~Under-5 deaths: β=0.94 (0.94, 0.94) and Maternal deaths: β=0.95 (0.92, 0.98)			
**Outdoor Air Pollution, Preterm Birth, and Low Birth Weight: Analysis of the World Health Organization Global Survey on Maternal and Perinatal Health; Fleischer et al. [31]**	2014	Democr atic Republi c of Congo, Kenya, Niger, Nigeria	Sept. 2004 – March 2005	Cross-sectional health facility study	N=192,900 with n=35,125 from SSA countries	Low birth weight and Preterm birth	Seasonally adjusted long-term average PM2.5 estimates estimated around study clinics (50-km buffers). Estimates derived from satellite data.	Generaliz ed estimating equations accountin g for clinic correlatio n in birth outcomes	PM2.5 (3rd Quartile vs. 1st Quartile) ~ low birth weight: OR=1.15 (1.02, 1.30)<br><br>PM2.5 (4th Quartile vs. 1st Quartile) ~ low birth weight: OR=1.15 (1.01, 1.32)	No	No	No
**Interaction between ambient pollutant exposure, CD14 (-159) polymorphism and respiratory outcomes among children**	2017	Durban, South Africa	3-weeks during differe nt season s (study year not given in	Prospective cohort at schools with questionnaire	N=71, Children ages 7 – 9 recruited from 7 different schools	Repeated measures of within-day variability in Lung function (FEV1)	Daily concentrat ions of NO2, NO, SO2, and PM from continuou s air monitorin g: NO and NO2 measured at 7	Linear multivaria te generalize d estimated equations to account for correlatio n of FEV1 within participant	No overall associations between pollutants and within-day lung function; Significant gene-environment interaction (CD14 polymorphisms) for NO and	Yes – By CD14 polymorphis m genotype	No	No

**in Kwazulu-Natal, Durban; Makamure et al. [21]**			public ation)				monitorin g sites; SO_2 at 16 sites (including all 7 schools); PM10 at 12 sites (and each school)		NO_2 on within-day lung function;			

[a] Due to the large number of statistical comparisons related to multiple day lag tests, it is impractical to detail each effect estimate size in this table and we have therefore simply indicated the direction of the statistically significant association. The reader is referred to the original article to obtain the effect estimate size.

Table S2. Tabular summary of notable studies that did not meet the inclusion criteria.

Title, Author, and Year published	Country and Study Population	Health Outcomes, Pollutant Measured, and Outdoor Air Pollution Exposure Proxies	Key Findings
Urban city transportation mode and respiratory health effect of air pollution: a cross-sectional study among transit and non-transit workers in Nigeria.; Ekpenyong et al.; 2012	Nigeria; N=168 adult participants from Uyo metropolis	Outcomes: Lung function impairment (FVC and FEV) – GOLD and NICE criteria<br><br>Pollutants Measured: None<br><br>Exposure Proxies: Transit workers (motorcyclists/taxi drivers) and civil servants	Motorcyclists showed the highest odds of lung function impairment<br><br>Uneducated and smoking transit workers who worked for more than a year and had frequent and lengthy trips had highest lung function impairment
Air pollution and respiratory disease in a tropical urban setting in Cotonou, Benin; Agodokpessi et al.; 2011	Cotonou, Benin; residents from 60 homes	Outcomes: Lung function spirometry, respiratory symptoms<br><br>Pollutants Measured: NO_2, SO_2, and CO<br><br>Exposure Proxies: Heavy vs. Light traffic	Spirometry abnormalities as well as respiratory symptoms significantly higher during heavy versus light traffic
Air pollution in urban area in Cotonou and Lokossa, Benin; Fourn & Fayomi; 2006	Cotonou and Lokossa, Benin, 400 taxi drivers	Outcomes: Intoxication symptoms (e.g., respiratory, lacrimation) and carboxyhemoglobin<br><br>Pollutants Measured: Benzene, CO<br><br>Exposure Proxies: Motorcycle drivers versus non-Motorcycle drivers	Motorcycle drivers accounted for 90% of intoxication cases, Hbco levels were abnormal in 66% of drivers

The Burden of Hypertension in an Oil- and Gas-Polluted Environment: A Comparative Cross-Sectional Study; Ezejimofor et al.;2016	Niger River Delta Region of Nigeria; N=2028 adults	Outcomes: Hypertension<br><br>Pollutants Measured: None<br><br>Exposure Proxies: Polluted versus non-Polluted areas	5-times higher odds of hypertension living in polluted area with increased risk of pollution effects with increasing age
Association between wheeze and selected air pollution sources in an air pollution priority area in South Africa: a cross-sectional study; Shirinde et al.;2014	Ekurhuleni Metropolitan Municipality, South Africa; N=3468 schoolchildren ages 13-14	Health Outcomes: respiratory indicators from ISAAC questionnaire<br><br>Pollutants Measured: None<br><br>Exposure Proxy: Frequency of trucks passing nearby home; mode of transportation to school	Wheeze ever, current wheeze, and current severe wheeze significantly associated with higher frequency of trucks passing by children's home
Allergic rhinitis, rhinoconjunctivitis and hayfever symptoms among children are associated with frequency of truck traffic near residences: a cross sectional study; Shirinde et al.;2015	Ekurhuleni Metropolitan Municipality, South Africa; N=3424 schoolchildren ages 13-14	Health Outcomes: rhinitis ever, current rhinitis rhinoconjunctivitis and hayfever from ISAAC questionnaire<br><br>Pollutants Measured: None<br><br>Exposure Proxy: Frequency of trucks passing nearby home mode of transportation to school	Current rhinitis and current rhinoconjunctivitis were significantly associated with higher frequency of trucks passing by children's home
Self-reported truck traffic on the street of residence and symptoms of asthma and allergic disease: a global relationship in ISAAC phase 3.; Brunekreef et al.; 2009	Five African countries; N= 16,914 schoolchildren ages 6-7 and 13-14 years	Health Outcomes: symptoms of asthma, rhinoconjunctivitis, and eczema from ISAAC questionnaire<br><br>Pollutants Measured: None<br><br>Exposure Proxy: Frequency of trucks passing nearby home	Odds ratios (95% confidence intervals) for current wheeze and almost the whole day versus never truck traffic were 1.35 (1.23-1.49) for 13- to 14-year-olds and 1.35 (1.22-1.48) for 6- to 7-year-olds
Meteorologically estimated exposure but not distance predicts asthma symptoms in schoolchildren in the environs of a petrochemical refinery: a cross-sectional study; White et al.; 2009	Cape Town, South Africa; N=2,361 school children ages 11-14 years living near petrochemical facility	Health Outcomes: Asthma symptoms from ISAAC questionnaire<br><br>Pollutants Measured: None<br><br>Exposure Proxy: Distance and meteorology (wind direction/speed) from petrochemical facility	Meteorological (but not distance) exposure estimates significantly associated with higher inhaler use and recent waking with wheeze and wheeze during resting.
